# Chemical Profiling, Antioxidant, Cytotoxic Activities and Molecular Docking Simulation of *Carrichtera annua* DC. (Cruciferae)

**DOI:** 10.3390/antiox9121286

**Published:** 2020-12-16

**Authors:** Enas E. Eltamany, Sameh S. Elhady, Haidy A. Ahmed, Jihan M. Badr, Ahmad O. Noor, Safwat A. Ahmed, Mohamed S. Nafie

**Affiliations:** 1 Department of Pharmacognosy, Faculty of Pharmacy, Suez Canal University, Ismailia 41522, Egypt; enas_mostafa@pharm.suez.edu.eg (E.E.E.); haidyabdelkader@gmail.com (H.A.A.); gehan_ibrahim@pharm.suez.edu.eg (J.M.B.); 2 Department of Natural Products, Faculty of Pharmacy, King Abdulaziz University, Jeddah 21589, Saudi Arabia; ssahmed@kau.edu.sa; 3 Ismailia Health Affairs Directorate, Ismailia 41525, Egypt; 4 Department of Pharmacy Practice, Faculty of Pharmacy, King Abdulaziz University, Jeddah 21589, Saudi Arabia; aonoor@kau.edu.sa; 5 Department of Chemistry, Faculty of Science, Suez Canal University, Ismailia 41522, Egypt; Mohamed_nafie@science.suez.edu.eg

**Keywords:** *Carrichtera annua*, LC-ESI-TOF-MS/MS, antioxidant, antiproliferative, docking studies

## Abstract

Our investigation intended to analyze the chemical composition and the antioxidant activity of *Carrichtera annua* and to evaluate the antiproliferative effect of *C. annua* crude and phenolics extracts by MTT assay on a panel of cancerous and non-cancerous breast and liver cell lines. The total flavonoid and phenolic contents of *C. annua* were 47.3 ± 17.9 mg RE/g and 83.8 ± 5.3 mg respectively. *C. annua* extract exhibited remarkable antioxidant capacity (50.92 ± 5.64 mg GAE/g) in comparison with BHT (74.86 ± 3.92 mg GAE/g). Moreover, the extract exhibited promising reduction ability (1.17 mMol Fe^+2^/g) in comparison to the positive control (ascorbic acid with 2.75 ± 0.91) and it displayed some definite radical scavenging effect on DPPH (IC_50_ values of 211.9 ± 3.7 µg/mL). Chemical profiling of *C. annua* extract was achieved by LC-ESI-TOF-MS/MS analysis. Forty-nine hits mainly polyphenols were detected. Flavonoid fraction of *C. annua* was more active than the crude extract. It demonstrated selective cytotoxicity against the MCF-7 and HepG2 cells (IC_50_ = 13.04 and 19.3 µg/mL respectively), induced cell cycle arrest at pre-G1 and G2/M-phases and displayed apoptotic effect. Molecular docking studies supported our findings and revealed that kaempferol-*3,7-O-bis-α-*L-rhamnoside and kaempferol-3-rutinoside were the most active inhibitors of Bcl-2. Therefore, *C. annua* herb seems to be a promising candidate to further advance anticancer research. In extrapolation, the intake of *C. annua* phenolics might be adventitious for alleviating breast and liver malignancies and tumoral proliferation in humans.

## 1. Introduction

At the moment, cancer is the second cause of mortality and morbidity worldwide [1,2]. It accounts for 9.6 million cases of deaths in 2018 as estimated by WHO [3,4]. Based on the incident rate, the most common neoplasms in women are breast, cervical, colorectal, thyroid and lung tumors. On the other hand, liver, prostate, colorectal, stomach and lung are the most common types of cancer in men [4]. The rapid creation and growth of abnormal cells in neoplasms is correlated to the un-controlled cell hyper-proliferation which has several hallmarks, such as resistance to apoptosis, insensitivity to antigrowth signals, enabling angiogenesis, activation of tissue invasion and metastasis [5,6,7]. Metastases are the major cause of death [5]. Due to the increase of cancer cases and consequently the rise in mortality rates, Cancer therapy is an ongoing challenge with better treatment protocols needed. Until now, surgery combined with radio and chemotherapies are the most impactful treatment approach. Regrettably, resistance to various chemotherapies and a lack of drug selectivity and toxicity are also problematic. Hence, there is an increasing need for new treatment strategies and more effectual antineoplastic agents to combat malignant tumors [2,3].

Natural products are promising candidates as anticancer agents for being more available and less harmful [1,2]. Another possibility is the combination of existing chemotherapeutics with plant polyphenols [3]. Polyphenols have emerged as one of the most abundant natural products with a relevant antioxidant activity therefore, they oppose ROS formation and attenuate oxidative DNA damage and mitochondrial dysfunction by acting as chemo-preventive agents. Moreover, several polyphenols have reported to induce cell cycle arrest in different malignant cell lines [1]. Plant derived flavonoids have been validated to be efficient chemotherapeutic candidates against numerous cancers via modulation of apoptosis. The main mechanisms involved are the intrinsic and extrinsic activation of apoptotic proteins, and induction of DNA damage besides their interference with multiple signal transduction in the process of carcinogenesis and consequently obstruct proliferation, angiogenesis and metastasis [1,2].

Family Brassicaceae (Cruciferae) is composed of 350 genera including about 3500 species [8]. Species of family Brassicaceae are considered as a good source of food, vegetable oils and spices [9]. Additionally, Family Brassicaceae accumulates different groups of phytochemicals such as phenolics [10,11], flavonoids [12], alkaloids [13] and glucosinolates [14]. These phytochemicals contribute to the reported antioxidant, antimicrobial, anti-inflammatory [15], anticancer [6], and cardiovascular protective activities [10]. *Carrichtera annua* L. is a plant belonging to family Brassicaceae. Despite several Brassicaceae species have been extensively subjected to phytochemical studies and investigations on their medicinal value in ameliorating human and animal diseases [16], there are limited reports concerning *C. annua*. Indeed, as far as we know, there are few studies on the chemical constituents of *C. annua* and a number of flavonoid derivatives were reported [17,18,19,20,21] and only article concerning the biological activity of *C. annua* where the anticomplement activity of the plant was reported [21]. On the basis of the aforementioned considerations, the present work involves the estimation of total phenolic and flavonoid contents, and antioxidant activity of *C. annua*. In addition, the whole plant is investigated for its chemical constituents using LC-ESI-TOF-MS/MS technique. Moreover, the antiproliferative effect of *C. annua* extract as well as its flavonoid rich fraction was assessed. The molecular docking tool was utilized to determine the most active compounds by inspecting their interaction with active cavities of the target receptors.

## 2. Materials and Methods 

### 2.1. Plant Material

*Carrichtera annua* (L.) DC Cruicferae (Brassicaceae) parts were collected from Sinai, Egypt. Authentication of the plant was done by Prof. Dr Elsayeda M. Gamal El-Din, Department of Botany, Faculty of Science, Suez Canal University. A voucher specimen of the plant was placed in the Herbarium of Pharmacognosy Department, Faculty of Pharmacy, Suez Canal University, Ismailia, Egypt (under registration number SAA-159). The collected plant was dried at room temperature and then pulverized.

### 2.2. Preparation of Plant Extract

Two hundred and fifty grams of powdered aerial part of *C. annua* were extracted with ethanol till exhaustion. The extracts were combined, dried under vacuum to give 10.94 g of brownish-green residue.

### 2.3. Determination of Total Phenolic Content

Quantification of total phenolics of the *C. annua* (L.) DC extract was achieved spectrophotometrically following the method described by the Saeed research team [22] with slight modification. A 200 μg/mL solution of the *C. annua* in methanol was prepared. The extract solution (0.5 mL) and 10-fold diluted Folin–Ciocalteu reagent (2.5 mL) were mixed together. Then 75 mg/mL sodium carbonate solution (2 mL) was added. The reaction mixture was kept for 10 min at 50 °C. Using gallic acid as a standard, the UV absorbance was recorded at λ 630 nm against blank using (Milton Roy, Spectronic 1201, Houston, TX, USA). The result was obtained as gallic acid equivalents (mg∙GAE/g dry extract)

### 2.4. Estimation of Total Flavonoid Content

Total flavonoids were estimated by AlCl_3_ method as mentioned by Saeed and coworker [22]. A solution of final concentration 1 mg/mL was prepared by dissolving the crude extract in methanol. 0.3 mL of the extract solution was diluted with 3.4 mL of methanol (30% *v*/*v*) and mixed with 0.15 mL 0.5 M NaNO_2_ and 0.3 M AlCl_3_.6H_2_O (0.15 mL of each). The mixture was subjected to vigorous shaking and incubated for 5 min. at 20 °C. Then 1 mL of 1M NaOH solution was added. By applying rutin as a standard, the UV absorption was recorded spectrophotometrically at λ 510 nm using (Milton Roy, Spectronic 1201, Houston, TX, USA). The result was obtained as rutin equivalent (mg∙RE/g dry extract).

### 2.5. Evaluation of Antioxidant Activity

#### 2.5.1. DPPH Free Radical Scavenging Activity

The free radical-scavenging activity of *C. annua* crude extract was evaluated by using the method reported by Fuochi and coworkers [23]. In short, a 100 µM solution of 2,2-diphenyl-1-picrylhydrazyl (DPPH) radical freshly prepared in methanol then stored at 10 °C in dark. The extract under investigation was prepared (at the various concentrations). An aliquot of 70 uL of the extract solution was added to DPPH solution (3 mL). The reaction mixture was incubated in dark for 30 min.at room temperature. The absorbance of the mixture was recorded at λ 515 nm with a UV-visible spectrophotometer (Milton Roy, Spectronic 1201, Houston, TX, USA). The control absorbance (only DPPH radical solution) and butylated hydroxytoluene (BHT) as a standard were also estimated. The measurements were calculated as the average of three replicates. The inhibition % (PI) of the DPPH radical was determined as previously reported from the formula:

PI = [{(*A*C−*A*T)/*A*C} × 100] (1)

where *A*C represents the control absorbance at and *A*T represents sample + DPPH absorbance.

The 50% inhibitory concentration (IC_50_) was calculated from the dose/response curve using Graphpad Prism software (San Diego, CA, USA).

#### 2.5.2. Ferric Reducing Antioxidant Power (FRAP) Assay

The FRAP of *C. annua* ethanol extract was determined using the procedure described by Nsimba in 2008 [24]. The mechanism of the assay based on electron transfer process at low pH, where the colourless complex (Fe^3+^-TPTZ) is reduced to the blue colored complex (Fe^2+^-tripyridyltriazine). The reaction was monitored by measuring the change in absorbance at λ 593 nm using (Milton Roy, Spectronic 1201, Houston, TX, USA). 40 µL of the extract solution was diluted with 0.2 mL of distilled water and mixed with 1.8 mL of warm freshly prepared FRAP reagent prepared as previously described [24]. The results were illustrated as the concentration of antioxidants having ferric reducing ability equivalent to that of 1 mM FeSO_4_, expressed as m Mol Fe^+2^ equivalent/g dry sample. The utilized positive controls were ascorbic acid and Butylated hydroxytoluene (BHT; Sigma-Aldrich, St. Louis, MO, USA).

#### 2.5.3. Total Antioxidant Capacity (TAC) Assay

The total extract of *C. annua* were evaluated for total antioxidant capacity (TAC) by spectrophotometrical determination using phosphomolybdenum assay. The procedure was executed as previously described by Saeed and his research team [22] with some modification. In Eppendorf tube; 0.1 mL of a 1 mg/mL extract (as methanolic solution) was added to reagent solution (1 mL). The tubes were capped, incubated in a water bath at 95 °C (for 90 min). The reaction mixture was then cooled to room temperature. Measurement of the absorbance was performed by UV-visible spectrophotometer using (Milton Roy, Spectronic 1201, Houston, TX, USA) at λ 695 nm against blank. TAC results were calculated (mg/g of dry sample) and expressed as gallic acid equivalents. Butylated hydroxytoluene (BHT; Sigma-Aldrich, St. Louis, MO, USA) was used as a reference compound.

### 2.6. Preparation of Phenolics Extract of C. annua 

Phenolic compounds of *C. annua* were extracted using the method described in El-Shaer and coworkers [25]. In brief, one hundred and fifty grams of powdered aerial parts of *C. annua* were treated with an aqueous solution of 5% Na_2_CO_3._ After one hour, the mixture was filtered and washed with distilled water to ensure complete extraction. The filtrate was diluted with distilled water and neutralized by HCl then partitioned between ethyl acetate and *n-*butanol. The obtained extracts were combined together then concentrated under reduced pressure to afford 4.03 g of *C. annua* total phenolics extract.

### 2.7. Preparation of the Sample and LC-HRMS Analysis

As previously described [26], the mobile phase working solution consisted of DI-Water: Acetonitrile: Methanol in a ratio of 50:25:25. Fifty mg of weighted dry methanolic extract was dissolved in one ml of MP-WS then vortex for 2 min. After that, ultra-sonication for 10 min followed by centrifugation for 10 min at 10,000 rpm were performed. 20 µL of the stock (50/1000 µL) was diluted with reconstitution solvent (1000 µL). At last, the concentration used for injection was 1 µg/µL where 25 µLs were injected in both positive and negative modes. Additionally, 25 µL of mobile phase working solution was used for injection as a blank sample. The used mobile phases consisted of: (A) 5 mM ammonium formate buffer pH 3 containing 1% methanol and used for positive TOF MS mode; (B) 5 mM ammonium formate buffer pH8 containing 1% methanol used for negative TOF MS mode; in addition to mobile phase (C) composed of 100% acetonitrile used for positive and negative modes. The pre column used was In-Line filter disks (Phenomenex, 0.5 µm × 3.0 mm) whereas, the column was X select HSS T3 (Waters, 2.5 µm, 2.1 × 150 mm) and the flow rate was 0.3 mL/min. Data processing was performed via MS-DIAL3.52. Feature (peaks) extraction from total ion chromatogram was achieved using Master view software, according to the following criteria: features intensities of the sample-to-blank should be more than 5 and features should have *Signal*-to-*Noise* not less than 5 (Non-targeted analysis). Identification of compounds was achieved via accurate mass measurements, MS/MS data, exploration of specific spectral libraries and public repositories for MS-based metabolomic analysis (MassBank NORMAN, MassBank MoNA, PubChem), retention times as well as data comparison with the literature. 

### 2.8. Biological Assays

#### 2.8.1. Cell Culture Treatment

A panel of cancerous and non-cancerous breast and liver cell lines; MCF-7, MDA_MB-231, MCF-10A, HepG2 and THLE2 were maintained in RPMI-1640/DMEM (Sigma-Aldrich, St. Louis, MO, USA). Both types of media were supplemented with 2mML-glutamine (Lonza, Belgium) and 10% FBS (Sigma, St. Louis, MO, USA), 1% Penicillin/Streptomycin (Lonza, Belgium). Incubation was performed for all cells at 37 °C in 5% CO2 atmosphere (NuAire, Lane Plymouth, MN 55447, USA) according to the routine tissue culture procedure [27].

#### 2.8.2. Cytotoxicity Using the MTT Assay

Cells were plated at a density of 5000 cells in triplicates in a 96-well plate. On the next day, treatment of the cells was performed with the indicated compound/s at the specified concentrations in 100 μL media as a final volume. Cell viability was considered after 72 h using MTT solution (Promega, Madison, WI, USA) [28]. The reagent (20 μLs) was added to each well followed by incubation of the plate for 3 h and fluorescence was subsequently measured (570 nm) using a plate reader, then IC_50_ values were assessed using the GraphPad prism 7 [29,30].

#### 2.8.3. Annexin V/PI and Cell Cycle Analysis

Apoptosis rate in cells was quantified using annexin V-FITC (BD Pharmingen, San Diego, CA, USA). Cells were seeded into 6-well culture plates (3 × 10^5^ cells/well); overnight incubation was done at 37 °C, under 5% CO_2_. Cells were then treated with indicated compounds for 48h. Next, media supernatants and cells were collected, followed by washing with ice-cold PBS. Next, cells were suspended in 100 µL of annexin binding buffer solution (25 mM CaCl_2_, 1.4 M NaCl, and 0.1 M Hepes/NaOH, pH 7.4). This was followed by incubation for 30 min in the dark with annexin V-FITC solution (1:100) and PI at 10 µg/mL concentration for 30 min. Stained cells were then acquired by Cytoflex FACS machin. Data was analyzed using cytExpert software. This assay was carried out as previously published in [31,32,33].

#### 2.8.4. RT-PCR

Treatment of MCF-7 cells with phenolics extract of *C. annua* (IC_50_ = 13.04 μg/mL) was done for 72 h. After completion of the treatment, collection of the cells and extraction of total RNA were performed using Rneasy^®^ Mini Kit (Qiagen, Hilden, Germany) as instructed by manufacturer. cDNA synthesis was executed using 500 ng of RNA using *i*-Script cDNA synthesis kit (BioRad, Hercules, CA, USA) according to the instructions of the manufacturer. Real-time RT-PCR reactions composed of 25 µL Fluocycle^®^II SYBR^®^ (Euroclone, Milan, Italy), 1.5 µL of both 10 µM forward and reverse primers, 3 µL cDNA, and 19 µL of H_2_O. Performance of the reactions was done for 35 cycles using temperature profiles as follows: for initial denaturation (95 °C for 5 m); for denaturation (95 °C for 15 min); Annealing (55 °C for 30 min), and finally extension (72 °C for 30 min) [31,32,33]. At the end, the Ct values were collected, and the relative folds of changes between all the samples. Primer used were listed in Table 1.

### 2.9. Simulated Molecular Docking 

All identified derivatives were screened for their binding activities towards the “B-cell lymphoma 2 (Bcl-2)” protein, its crystal structure with the co-crystallized ligand was freely accessible from the protein data bank. Processes concerning preparation of the protein, optimization of the ligands and software validation are implemented following the regular work as published by Nafie et al., 2019 [34]. The “MOE-2019 software” was used for molecular docking study. Each ligand-receptor complex was tested for binding interaction analysis with the binding energy (Kcal/mol), 3D images were performed using Chimera as a visualizing software [35].

## 3. Results and Discussion

### 3.1. Total Phenolic and Flavonoid Content

**Figure 1:**
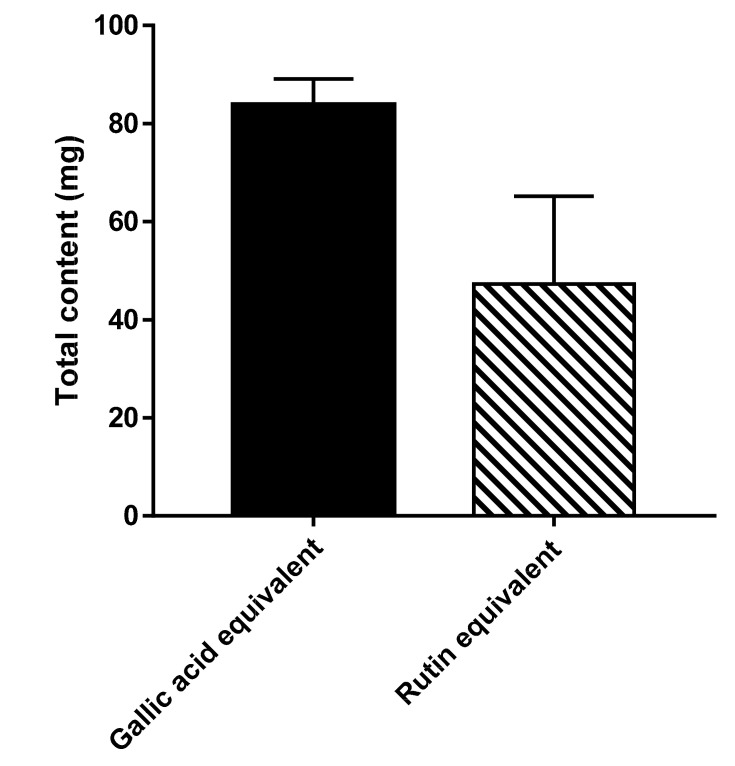
*C. annua* total phenolics contents (TPC) expressed as gallic acid equivalent per gram of dry weight GAE/g dw (83.8%) and total flavonoids contents (TFC) expressed as rutin equivalent per gram of dry weight RE/g dw (47.3%).

Phenolic compounds are widespread phytoconstituents and their main sources in human diet are fruits and vegetables. The same bioactive polyphenols, such as hydroxycinnamic acid derivatives, flavonoids and proanthocyanidins are also obtainable from forest trees [22]. Therefore, determination of the polyphenols content in the extract; the total phenols and total flavonoids is reasonable, in order to estimate the potential antioxidant capacity of *C. annua* crude extract. Total phenolic content of *C. annua* extract was estimated spectrophotometrically using Folin–Ciocalteu reagent. Based on the calibration curve of gallic acid, the obtained linear equation obtained was Y= 0.0011X + 0.0131 (R^2^ = 0.9946). The total phenolic content of *C. annua* methanolic extract was 83.8 ± 5.3 mg GAE/g of plant extract. Total flavonoid content in *C. annua* extract was obtained spectrophotometrically using AlCl_3_ reagent and rutin as standard. Derived from the calibration curve of rutin, the obtained linear equation was; Y = 0.0011X + 0.0131 (R^2^ = 0.9946). The total flavonoids content of *C. annua* methanolic extract estimated from the above equation was 47.3 ± 17.9 mgRE/g of plant extract (see Figure 1).

### 3.2. Evaluation of In Vitro Antioxidant Activity of C. annua Extract

**Figure 2:**
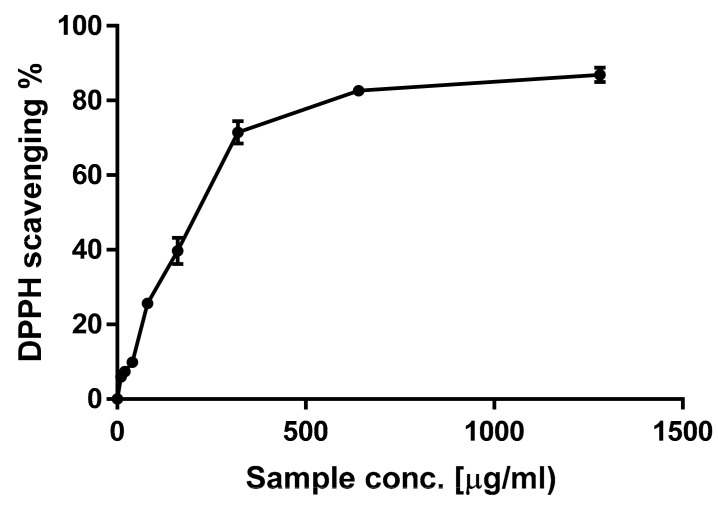
The scavenging rate (%) of 2,2-diphenyl- 1-picrylhydrazyl (DPPH) by *C. annua* crude extract. All of the values in the figure are expressed as means (%) and SD of triplicated experiments.

**Figure 3:**
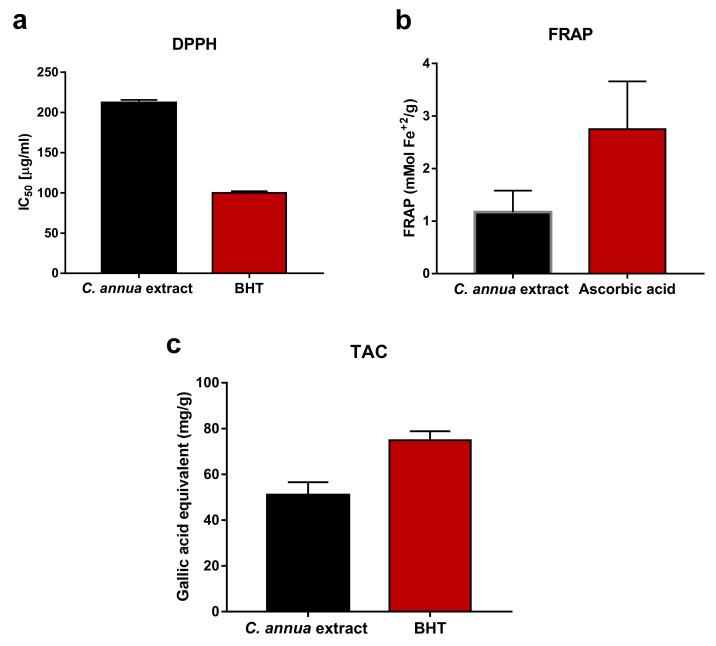
Antioxidant activity of *C. annua* crude extract (**a**) The IC_50_ value of 2,2-diphenyl-1-picrylhydrazyl (DPPH) radical scavenging assay, (**b**) ferric-ion reducing antioxidant power (FRAP) assay and (**c**) total antioxidant capacity (TAC) assay.

Flavonoids and phenolic acids are characterized by being electron or hydrogen donors, reducing and metal chelating agents. These properties arise from different conjugations and varying numbers of hydroxyl groups in their structures [16]. Therefore, due to the complexity of natural phytoconstituents and different scavenging modes of ROS (reactive oxygen species), a group of assays were used simultaneously in order to judge the antioxidant activity of *C. annua* extract, [36]. In the present study, three indicative tests (DPPH, FRAP, TAC) were applied to analyze the antioxidant power of *C. annua crude* extract. Figure 2 demonstrates that the crude extract had definite scavenging activity on DPPH exhibiting a dose-dependent scavenging rate. As shown in Figure 3a, *C. annua* crude extract with IC_50_ values of 211.9 ± 3.7 µg/mL revealed notable activities in DPPH radical scavenging assay compared to the positive control (BHT IC_50_ = 100 ± 2.1 µg/mL.). Results of FRAP (Figure 3b) demonstrates that *C. annua* had promising reduction ability with 1.17 mMol Fe^+2^ /g in comparison to the positive control (Ascorbic acid with 2.75 ± 0.91 respectively). Figure 3c demonstrates the total antioxidant capacity of *C. annua* extract and BHT (standard synthetic antioxidant) assessed by phosphomolybdenum test. *C. annua* extract exhibited remarkable antioxidant potential (50.92 ± 5.64 mg GAE/g) in comparison with BHT (74.86 ± 3.92 mg GAE/g).

### 3.3. LC-ESI-TOF-MS/MS Analysis

Herein, *Carrichtera annua* was proven to be a rich source of phenolics and flavonoids (83.8 ± 5.3 mg GAE/g and 47.3 ± 17.9 mg RE/g respectively). It demonstrated promising antioxidant potential by exhibiting notable free radical scavenging activity (IC_50_ = 211.9 ± 3.7 µg/mL) and ferric reduction power (17 mMol Fe^+2^/g) as well as good antioxidant potential (50.92 ± 5.64 mg GAE/g). As a consequence, *C. annua* crude extract was investigated by LC-ESI-TOF-MS/MS (Agilant, Santa Clara, CA, USA) in order to fully understand the chemical diversity of its phytoconstituents including phenolics and other metabolites accountable for the estimated antioxidant activity of the plant. Data are represented in Table 2 and LC-ESI-TOF-MS/MS profile is shown in (Supplementary Materials Figures S1–S4). Tentative identification of the individual components was achieved by comparison of their chromatographic behavior, *m*/*z* values in the total ion chromatogram (TIC) and base peak chromatogram (BPC) profile as well as their fragmentation pattern with those described in the literature.

**Figure 4:**
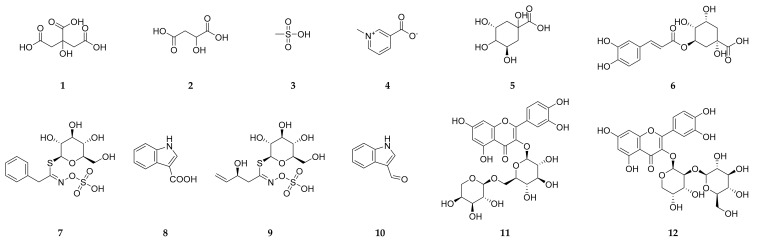

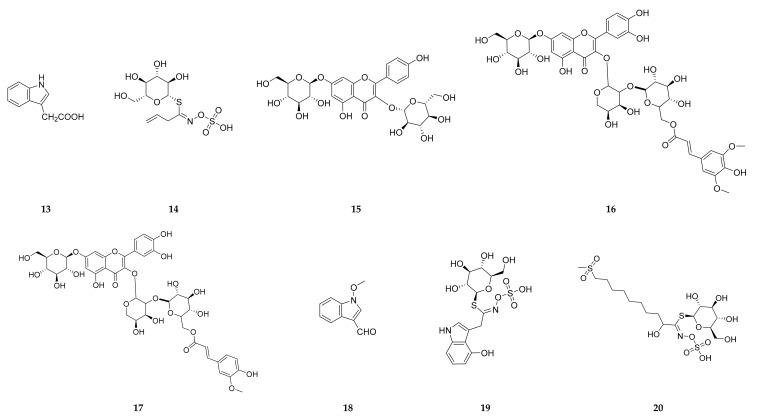

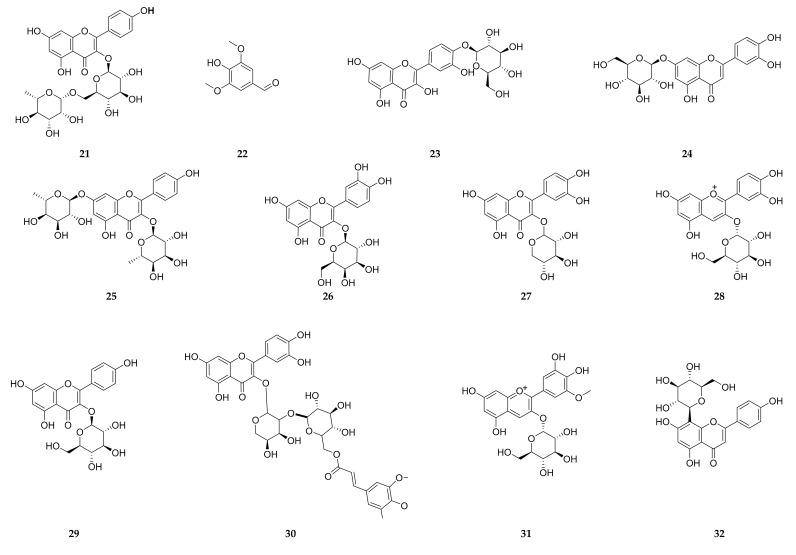

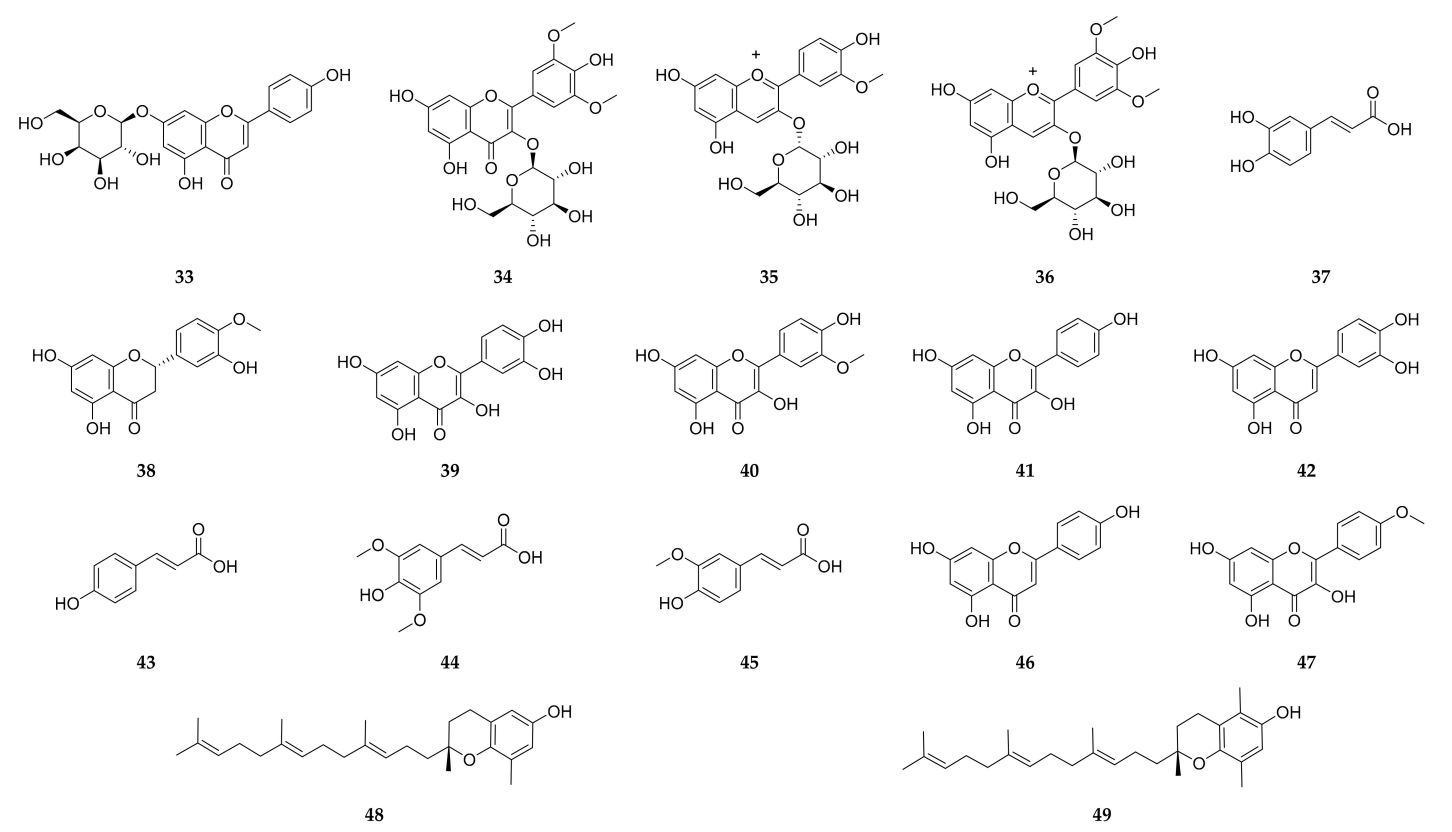
Chemical structures of the identified compounds by LC-ESI-TOF-MS/MS.

In particular, 49 hits were identified in *C. annua* (Table 2, Figure 4) belonging to different metabolic classes; mainly phenolics. Fifteen flavonol derivatives have been detected in *C. annua* extract among which quercetin-*3*-*O*-arabinoglucoside (peltatoside), quercetin *3-O-β*-D-glucopyranosyl-(1–>2)-arabinopyranoside, quercetin *3-O*-[(6 sinapoyl-*β*-glucopyranosyl)(1–>2)-*β*-arabinopyranoside]-*7-O-β*-glucopyranoside, quercetin *3-O*-[(6-feruloyl-*β*-glucopyranosyl)-(1–>2)-*β*-arabinopyranoside]-*7-O-β*-glucopyranoside, quercetin *3-O*-[(6-sinapoyl-*β*-glucopyranosyl)-(1–>2)-*β* arabinopyranoside, kaempferol-3 rutinoside and isorhamnetin have been reported previously in *C. annua* [17,19,20,21]. It is noteworthy to mention that, despite of quercetin-*7-O*-arabinosyl-*3-O* glucoside and quarecetin-*3-O*-glucoside [21] were isolated from *C. annua*, they were not detected in our extract, instead we recorded quercetin-*4′-O-*glucoside. In addition to flavonols, luteolin, and apigenin flavone aglycones and glycosides were recorded in the present study. Moreover, four anthocyanins (cyanidin-3-glucoside, petunidin-*3-O-β*-glucopyranoside, peonidine-*3-O*-glucoside and malvidin-3-galactoside) have been reported for the first time in *C. annua.* Ferulic, *p* coumaric, caffeic and sinapic acids were detected along with other organic acids.

Glucosinolates (GSs) are sulfur- and nitrogen-containing compounds widely distributed in Brassicaceae plants [37]. Five GSs (progoitrin, sinigrin, glucotropaeolin, 4-hydroxyglucobrassicin and 9-(methylsulfonyl) hydroxy nonyl glucosinolate) were detected in the current study. On the other hand, gluconapin, brassicanapin, erucin, glucoberteroin, glucoerucine and glucoraphanin which were reported in Spanish and Australian *C. annua* [38,39] were not detected. 

Alkaloids are nitrogenous compounds synthesized from amino acids and have been reported in several Brassicaceae plants [13]. In the present investigation, four nitrogenous compounds particularly of indole type (1H-indole-3-carboxylic acid, 3-formyl indole, 2-(1H-indol-3-yl) acetic acid and 1-methoxy-1H-indole-3-carbaldehyde) which were previously reported in *Isatis tinctoria*; a plant belongs to family Brassicaceae [40] were also recorded in this study. No tropane alkaloids were detected in this study which coincide with Brock and coworker [41]. However, our findings went against them in the presence of calystegines since there were none of them were reported. Interestingly, trigonelline was recorded for the first time in a member of family Brassicaceae.

Tocotrienols and tocopherols are naturally occurring terpenes present in Brassicaceae plants and have diverse biological activities [13]. Our results revealed the presence of *δ*- tocotrienol and *β*-tocotrienol in *C. annua*.

### 3.4. Biology

#### 3.4.1. Cytotoxicity Using the MTT Assay

**Figure 5:**
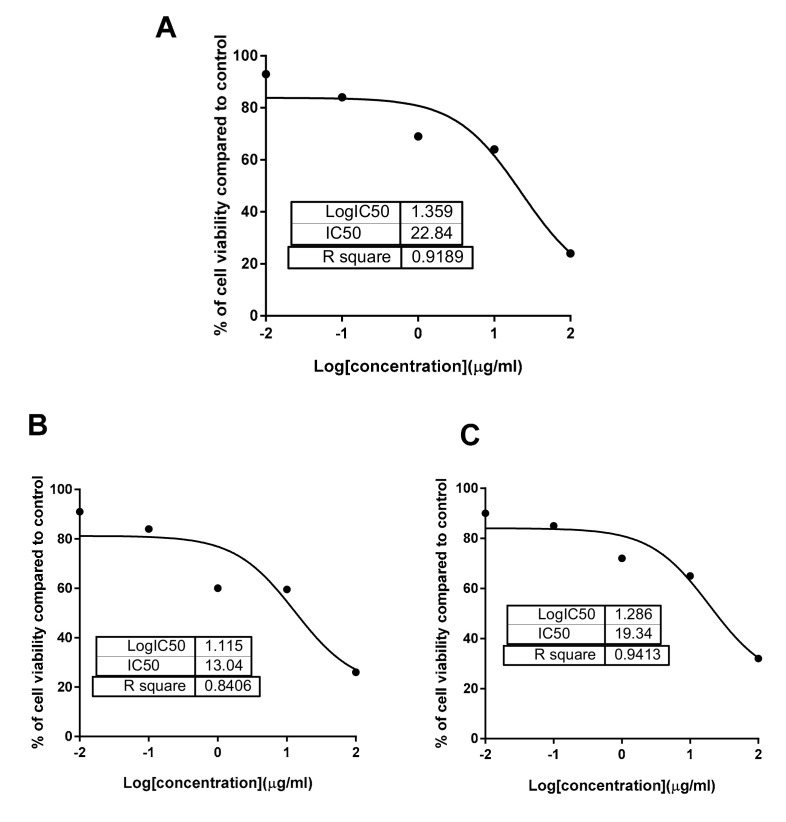
IC_50_ nonlinear regression curve fit of percentage of cell viability vs log [con. µg/mL], R square ≈1, using the GraphPad prism software. (**A**) Cytotoxicity of crude extract against MCF–7, (**B**) Cytotoxicity of phenolics extract against MCF–7, (**C**) Cytotoxicity of phenolics extract against HepG2.

Evidence of the anticancer activities of family Brassicaceae plants against various types of malignancies have been acquired in numerous biological investigations [16,75,76,77,78,79]. These antitumor activities are mediated by different mechanisms such as antioxidant, cell cycle arrest, induction of apoptosis and prevention of angiogenesis and metastasis [80,81]. Candidate phytoconstituents responsible for these antineoplastic properties are glucosinolates and their hydrolytic products and phenolics (particularly flavonoids) as well [78]. Hence, the crude and phenolics extracts of *C. annua* were screened for their cytotoxic activities against panel of cancerous and non-cancerous breast and liver cell lines; MCF-7, MDA-MB-231, MCF-10A, HepG2 and THLE2, and to test their safety (selectivity) using the MTT assay (Table 3). *C. annua* crude extract was most cytotoxic against the MCF-7 cell line (IC_50_ = 22.8 µg/mL) with cell growth inhibition 25.4% at the highest concentrations 100 µg (Figure 5), while it was selective against the MDA-MB-231 with higher IC_50_ value of 46.2 µg/mL, and safe against normal breast and liver cells.

On the other hand, phenolics extract of *C. annua* was much more cytotoxic against the MCF-7 cells (IC_50_ = 13.04 µg/mL) than the crude extract, additionally, it showed more cytotoxic activity against the HepG2 cells (IC_50_ = 19.3 µg/mL) than the crude extract. On the other hand, it was not toxic against other cells, which elucidates the selectivity of its action. So, these results indicated the activity of phenolics extract against MCF-7 and HepG2 cells in a selective way for the other cells. Hence, phenolics extract was assumed of value to be investigated to determine its impact on induction of apoptosis in both MCF-7 and HepG2 cancer cells.

#### 3.4.2. Annexin V/PI and Cell Cycle Analysis

**Figure 6:**
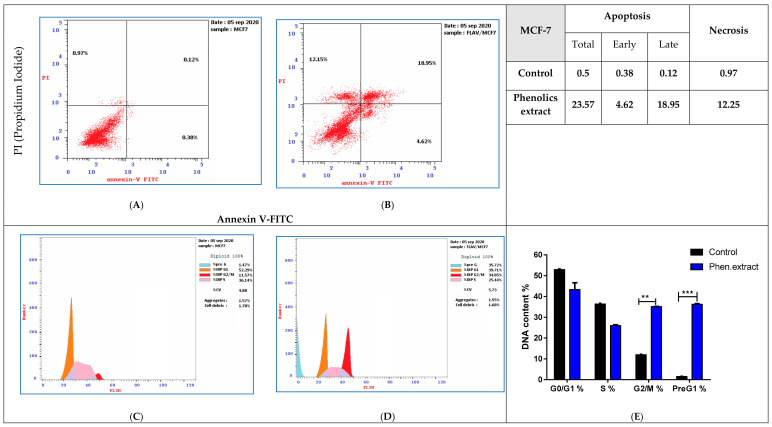
FITC/Annexin-V-FITC/PI differential apoptosis/necrosis (**A**) untreated control, (**B**) Phenolics extract (IC_50_ = 13.04 μg/mL, 48 h) and DNA content-flow cytometry aided cell cycle analyses (**C**) untreated control, (**D**) Flavonoid extract (IC_50_ = 13.04 μg/mL, 48 h), (**E**) bar chart representation) in MCF–7. ** *p* ≤ 0.05 and *** *p* ≤ 0.001 are significant different.

Treatment of MCF-7 cancer cells with phenolics extract (IC_50_ = 13.04 μg/mL, 48 h) was performed. Investigations were done for its apoptosis-inducing activity using the cell cycle analysis with the cell population in different cell cycle phases. Investigation of the cell cycle is a decisive test that declares the cell accumulation percentage in each growth phase with cytotoxic substances after treatment. As shown in Figure 6 (upper panel), phenolics extract remarkably stimulated apoptotic breast cancer cell death with 47.14-fold (23.57% compared to 0.51% for the control). It induced early apoptosis by 12.15-fold (4.62% compared to 0.38% for control), and late apoptosis by 157.9-fold (18.95% compared to 0.12% for control). While it stimulated cell death via necrosis with 12.63-fold (12.25%, compared to 0.97% for the control). Moreover, MCF-7 cancer cells after phenolics extract treatment were subjected to DNA flow cytometry to analyze the cell cycle kinetics to determine the compound’s phase interference with the cell cycle. As seen in Figure 6C–E. It increased G2/M cell (34.85%, compared to 11.57% for control), and pre-G1 (35.72%, compared to 1.47% for the control) population, also it reduced cell number in the S (25.44% compared to 36.14% for control). 

**Figure 7:**
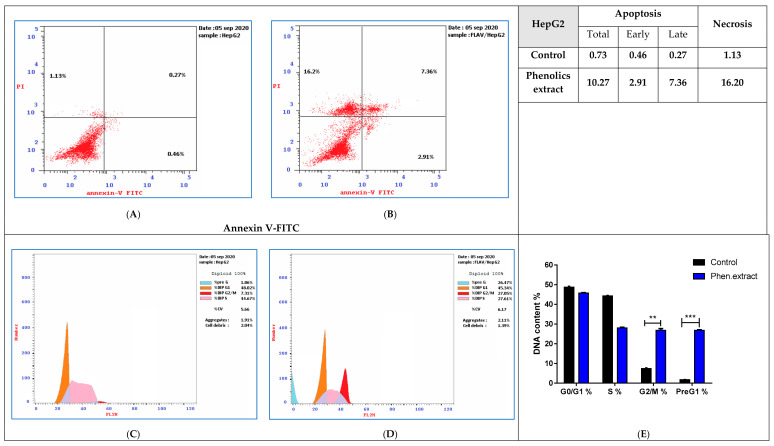
FITC/Annexin-V-FITC/PI differential apoptosis/necrosis (**A**) untreated control, (**B**) Phenloics extract (IC_50_ = 19.34 μg/mL, 48 h) and DNA content-flow cytometry aided cell cycle analyses (**C**) untreated control, (**D**) Flavonoid extract (IC_50_ = 13.04 μg/mL, 48 h), (**E**) bar chart representation in HepG2 cells. ** *p* ≤ 0.05 and *** *p* ≤ 0.001 are significant different

Similarly, HepG2 cancer cells were remedied with phenolics extract (IC_50_ = 19.34 μg/mL, 48 h). As seen in Figure 7 (upper panel), phenolics extract remarkably stimulated apoptotic breast cancer cell death with 14.07-fold (10.27% while it was 0.73% for the control). It induced early apoptosis by 6.32-fold (2.91% compared to 0.46% for control), and late apoptosis by 27.25-fold (7.36% compared to 0.27% for control). Moreover, HepG2 cancer cells after phenolics extract treatment were subjected to DNA flow cytometry, as seen in Figure 7 (lower panel). It enhanced G2/M cell (27.05%, compared to 7.3% for control), and pre-G1 (26.47%, compared to 1.86% for the control) population, also it reduced cell population in the S (27.61% compared to 44.67% for control).

Consequently, phenolics extract induced pre-G1 and G2/M-phase cell cycle arrest and blocked the progression of MCF-7 and HepG2 cancer cells that deteriorate the genetic metrical.

#### 3.4.3. RT-PCR Analysis

For investigation the apoptotic pathway for the phenolics extract, 13.2 μg/mL of the sample was added to MCF-7 cells and left for 72 h to allow complete interaction, after RNA extraction, cDNA was produced. Then the expression of mRNA of Caspases 3, 8 9, pro-apoptotic (P53, BAX, PUMA) as well as anti-apoptotic genes (Bcl-2) in MCF-7 cells was traced by the RT-PCR analysis.

**Figure 8:**
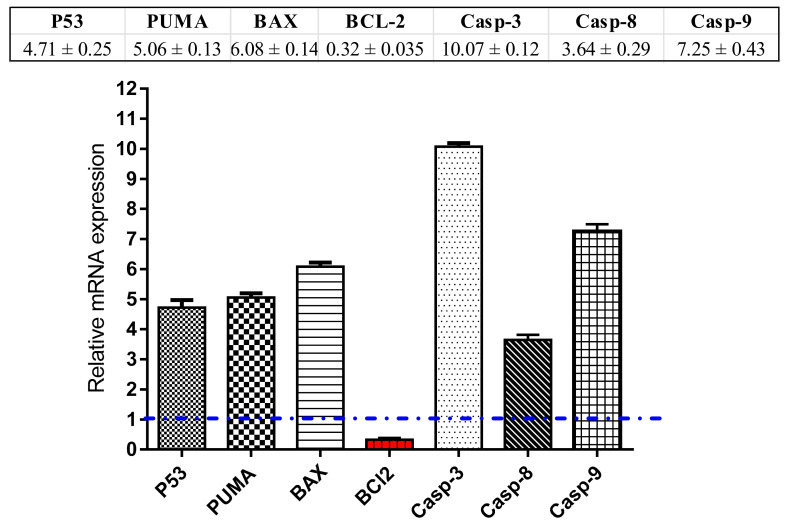
RT-PCR analysis of the apoptosis-related genes was performed after the MCF-7 cells were treated with phenolics extract (13.04 μg/mL) for 72 h.

As demonstrated in Figure 8, the expression of P53 gene was noticeably elevated by the phenolics extract (≈4.7-fold) with concomitant activation of the PUMA and BAX levels which have been raised by ≈5.06-fold and 6.08-fold, respectively. Furthermore, flavonoid extract has remarkably raised the mRNA expression of caspases 3, 8, 9 genes by ≈10.7-fold, 3.64-fold and 7.25-fold, respectively. On the hand, it markedly suppressed the expression of Bcl-2 (the anti-apoptotic gene) by ≈0.32-fold. These findings are in harmony with the apoptotic mechanism suggested for anti-cancer activity.

### 3.5. Simulated Molecular Docking Experiment

**Figure 9:**
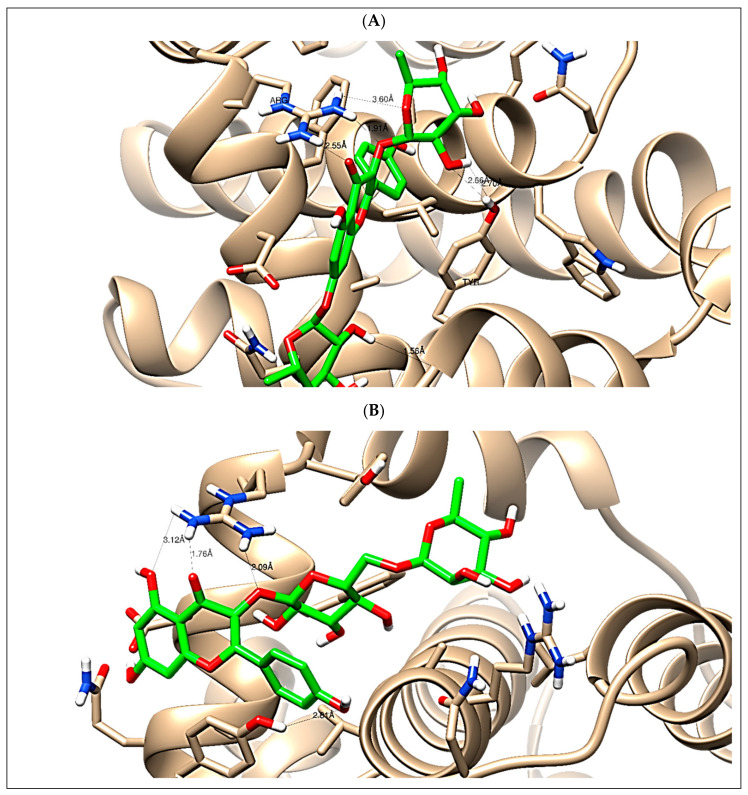
Visualized docked compounds: (**A**) Kaempferol-3,7-*O-bis-α*-L-rhamnoside **(25)** and (**B**) Kaempferol-3-rutinoside **(21)** inside the “B-cell lymphoma 2 (Bcl-2) (PDB ID: 4IEH) representing key interactions with bond length (Å) with interactive amino acids (Arg 66 and Tyr 161).

In this present study, *C. annua* phenolics extract exhibited antiproliferative activity against breast and liver carcinomas via apoptosis. Hence, the phenolics and flavonoids identified by the present LC-ESI-TOF-MS/MS analysis were selected for a simulated molecular docking investigation to gain insights into the possible molecular targets for the cytotoxic and apoptosis-inducing activities. The majority of the identified compounds revealed good binding interactions with binding energies (−9.24 to −27.28 Kcal/mol) inside the “B-cell lymphoma 2 (Bcl-2) (PDB ID: 4IEH) and their full interactions were summarized in Table 4. Other minor compounds didn’t show any binding activity towards the studied target. Accordingly, our docking experiment proposed their mechanism of action as Bcl-2 suppressors which is congruent with the flow cytometric and the RT-PCR analyses illustrating the apoptosis-inducing activity. As seen in Figure 9, Kaempferol-*3,7-O-bis-α-*L-rhamnoside and Kaempferol-3-rutinoside formed the maximum interactions with the interactive amino acids Arg 66 and Tyr 161 with binding energies of −23.67 and −18.28 (Kcal/mol), as they formed three hydrogen bonds with Arg 66 amino acid.

The current study has highlighted the noteworthy antioxidant and anticancer activities of *C. annua.* Therefore, this plant could be beneficial in cancer chemo preventive and chemotherapy. It represents a promising candidate to be used in combination with the existing chemotherapies. This combination tends to overcome drug resistance, increase the sensitivity to chemotherapy and counteract their dose dependent side effects especially those arise from the increased oxidative stress such as cardio and nephro toxicities. A future pharmacological study will be conducted to assess the in vivo efficacy and safety of *C. annua* as an antitumor herb individually and in combination with conventional chemotherapies to verify our assumption.

## 4. Conclusions

*C. Annua* herb can be considered as a promising chemo preventive and anticancer plant owing to its antioxidant and anti-proliferative effects that attributed to its unique chemical constituents. Herein, *C. annua* herb was studies for the first time for its chemical profiling and anticancer potential as well. Using LC-ESI-TOF-MS/MS analysis, 49 hits were identified mainly of polyphenolic type where flavonoid derivatives predominated and some of them were recorded in the plant for the first time. On the other hand, both of *C. annua* crude extract and its flavonoid fraction displayed significant and selective anticancer activity on HepG2 and MCF-7 cancer cells. However, the phenolic fraction was more active than the extract and it induced cell cycle arrest at pre-G1 and G2/M-phases by activation of pro apoptotic proteins and suppression of anti-apoptotic ones. The molecular docking studies indicated that most of the polyphenolics identified in the *C. annua* extract exhibited good binding interactions with binding energies (−9.24 to −27.28 Kcal/mol) inside the “B-cell lymphoma 2 (Bcl-2). Both of kaempferol-*3,7-O-bis-α-*L-rhamnoside and kaempferol-3-rutinoside were the most active inhibitors of Bcl-2. Therefore, *C. annua* herb seems to be a promising candidate to further advance anticancer research. In extrapolation, the intake of *C. annua* phenolics might be adventitious for alleviating breast and liver malignancies and tumoral proliferation in humans.

## Figures and Tables

**Table 1:** Primers used for real-time RT-PCR.

Primer	Sequence
β-Actin	FOR: 5’-GCACTCTTCCAGCCTTCCTTCC-3’ REV: 5’-GAGCCGCCGATCCACACG-3’
P53	FOR: 5’-CTTTGAGGTGCGTGTTTGTG-3’ REV: 5’-GTGGTTTCTTCTTTGGCTGG-3’
Bcl-2	FOR: 5’-GAGGATTGTGGCCTTCTTTG-3’ REV: 5’-ACAGTTCCACAAAGGCATCC-3’
PUMA	FOR: 5’-GAGGAGGAACAGTGGGC-3’ REV: 5’-CTAATTGGGCTCCATCTCGG-3’
BAX	FOR: 5’-TTTGCTTCAGGGTTTCATCC-3’ REV: 5’-CAGTTGAAGTTGCCGTCAGA-3’
Casp-3	FOR: 5’-TGGCCCTGAAATACGAAGTC-3’ REV: 5’-GGCAGTAGTCGACTCTGAAG-3’
Casp-8	FOR: 5’-AATGTTGGAGGAAAGCAAT-3’ REV: 5’-CATAGTCGTTGATTATCTTCAGC-3’
Casp-9	FOR: 5’-CGAACTAACAGGCAAGCAGC-3’ REV: 5’-ACCTCACCAAATCCTCCAGAAC-3’

**Table 2:** Metabolites identified in *C. annua* crude extract using LC-ESI/TOF/MS/MS.

Compound No	Rt (Min.)	Proposed Compound	Molecular Formula	Precursor Type	Calcd. *m*/*z* for Precursor	Obs. *m*/*z* for Precursor	MS/MS	Ref.
**1**	1.05	Citric acid	C_6_H_8_O_7_	[M−H]^−^	191.0192	191.0193	173, 111	[42]
**2**	1.18	Malic acid	C_4_H_6_O_5_	[M−H]^−^	133.0137	133.0141	155, 71	[42,43]
**3**	1.24	Methanesulfonic acid	CH_4_O_3_S	[M−H]^−^	94.9803	94.9807	80	[44]
**4**	1.39	Trigonilline	C_7_H_7_NO_2_	[M+H]^+^	138.0550	138.0551	94, 92	[45]
**5**	1.44	D-(-)-Quinic acid	C_7_H_12_O_6_	[M−H]^−^	191.0556	191.0556	173, 147, 85	[46,47]
**6**	2.02	Caffeoyl-quinic acid	C_16_H_18_O_9_	[M−H]^−^	353.0872	353.0868	191, 179, 135	[42]
**7**	2.23	Glucotropaeolin	C_14_H_19_NO_9_S_2_	[M−H]^−^	408.0423	408.0268	408, 162, 195, 246, 228	[48]
**8**	2.98	1H-indole-3 carboxylic acid	C_9_H_7_NO_2_	[M−H]^−^	160.0399	160.0399	116, 142, 143	[40,49]
**9**	3.22	Progoitrin	C_11_H_19_NO_10_S_2_	[M−H]^−^	388.0372	388.0370	388, 274, 259, 210, 192	[50,51]
**10**	4.18	3-formylindole	C_9_H_7_NO	[M+H]^+^	146.0606	146.0608	118	[40,49]
**11**	4.60 *	Quercetin-*3-O*-arabinoglucoside	C_26_H_28_O_16_	[M−H]^−^	595.1299	595.1303	462, 433, 301, 300, 299	[21,52]
**12**	4.68 *	Quercetin *3-O-β*-D-glucopyranosyl-(1→2)-arabinopyranoside	C_26_H_28_O_16_	[M−H]^−^	595.1299	595.1306	301, 311, 433	[19,52]
**13**	4.75	2-(1H-indol-3-yl) acetic acid	C_10_H_9_NO_2_	[M+H]^+^	176.0712	176.0736	176, 159, 158 130, 118	[40,49]
**14**	5.10	Sinigrin	C_10_H_17_NO_9_S_2_	[M−H]^−^	358.0266	358.0569	358, 278, 275, 259, 241	[50]
**15**	5.29	Kaempferol 3, 7 di-glucoside	C_27_H_30_O_16_	[M−H]^−^	609.1456	609.1460	489, 447, 285	[42]
**16**	5.69	Quercetin*3-O*- [(6 sinapoyl-*β*-glucopyranosyl) (1→2)-*β*-arabinopyranoside]-*7-O-β*-glucopyranoside	C_43_H_48_O_25_	[M−H]^−^	963.2406	963.2443	801, 595, 463, 385	[17,20,42]
**17**	5.70	Quercetin*3-O*-[(6-feruloyl-*β*-glucopyranosyl) -(1→2)-*β*-arabinopyranoside]-*7-O-β*-glucopyranoside	C_42_H_46_O_24_	[M−H]^−^	933.2301	933.2311	771, 739, 301	[17,18,42]
**18**	5.93	1-methoxy-1H-indole-3-carbaldehyde	C_10_H_9_NO_2_	[M+H] ^+^	176.0712	176.0749	161, 133, 117	[40,49]
**19**	6.09	4-Hydroxyglucobrassicin	C_16_H_20_N_2_O_10_S_2_	[M−H]^−^	463.0481	463.1205	463, 291, 275, 259, 241, 195	[48,53]
**20**	6.46	9(methylsulfonyl)hydroxy nonyl glucosinolate	C_17_H_33_NO_12_S_3_	[M−H]^−^	538.1087	538.8553	259, 275, 291	[37]
**21**	6.46	Kaempferol-3 rutinoside	C_27_H_30_O_15_	[M−H]^−^	593.1506	593.1503	285, 447	[19,42]
**22**	6.66	Syringaldehyde	C_9_H_10_O_4_	[M−H]^−^	181.0501	181.0511	181, 151	[54]
**23**	6.84	Quercetin-4’-glucoside	C_21_H_20_O_12_	[M+H]^+^	465.1033	465.1028	465, 303	[55]
**24**	6.91	Luteolin-7-O-glucoside	C_21_H_20_O_11_	[M−H]^−^	447.0927	447.09366	447, 285	[56]
**25**	6.94	Kaempferol-*3,7-O-bis-α-*L-rhamnoside	C_27_H_30_O_14_	[M−H]^−^	577.1557	577.1576	431, 285	[57]
**26**	6.95	Quercetin *3-O*-galactoside	C_21_H_20_O_12_	[M+H]^+^	465.1033	465.1049	465, 303	[58]
**27**	6.97	Quercetin-3-D-xyloside	C_20_H_18_O_11_	[M−H]^−^	433.0771	433.0772	301, 300, 271, 151	[52]
**28**	6.99 *	Cyanidin-3-glucoside	C_21_H_21_O_11_	[M]^+^	449.1084	449.1075	449, 287	[59,60,61]
**29**	7.00 *	Kaempferol-3-*O* glucoside	C_21_H_20_O_1_	[M+H]^+^	449.1084	449.1085	449, 287	[58,62]
**30**	7.09	Quercetin *3-O*-[(6-sinapoyl-*β*-glucopyranosyl)-(1→2)-*β* arabinopyranoside	C_37_H_38_O_20_	[M−H]^−^	801.1878	801.1855	801, 595, 300	[17]
**31**	7.23	Petunidin-*3-O-β-*glucopyranoside	C_22_H_23_O_12_	[M]^+^	479.1190	479.116	317, 302	[63]
**32**	7.75	Vitexin	C_21_H_20_O_10_	[M−H]^−^	431.0978	431.0989	431, 311, 283,	[64]
**33**	7.77	Cosmosiin	C_21_H_20_O_10_	[M+H]^+^	433.1135	433.1154	433, 271	[65]
**34**	7.79	Syringetin-*3-O*-glucoside	C_23_H_24_O_13_	[M−H]^−^	507.1139	507.1134	507, 345, 179	[66]
**35**	7.97	Peonidine-*3-O*-glucoside	C_22_H_23_O_11_	[M]^+^	463.1240	463.1223	463, 301, 286	[59,60]
**36**	8.14	Malvidin-3-galactoside	C_23_H_25_O_12_	[M]^+^	493.1346	493.1358	493, 331	[59,60]
**37**	8.31	Caffeic acid	C_9_H_8_O_4_	[M−H]^−^	179.0344	179.0343	179, 151, 136, 133	[67]
**38**	8.69	Hesperetin	C_16_H_14_O_6_	[M−H]^−^	301.0712	301.0711	301, 283, 271, 258	[68]
**39**	8.72	Quercetin	C_15_H_10_O_7_	[M+H]^+^	303.0505	303.0457	303, 153	[69]
**40**	9.13	Isorhamnetin	C_16_H_12_O_7_	[M−H]^−^	315.0505	315.0513	315, 300, 151	[20,70]
**41**	9.39	Luteolin	C_15_H_10_O_6_	[M−H]^−^	285.0399	285.0402	285, 267, 257, 241, 223, 197,175	[64,71]
**42**	9.40	Kaempferol	C_15_H_10_O_6_	[M−H]^−^	285.0399	285.0392	285, 257, 241, 223, 197, 151	[64,71]
**43**	9.60	*P* -coumaric acid	C_9_H_8_O_3_	[M−H]^−^	163.0395	163.0389	163, 119	[72]
**44**	9.88	Sinapic acid	C_11_H_12_O_5_	[M−H]^−^	223.0606	223.0964	223, 208, 179, 164	[42]
**45**	10.23	Ferulic acid	C_10_H_10_O_4_	[M−H]^−^	193.0501	193.0503	193, 178, 149	[42]
**46**	10.68	Apigenin	C_15_H_10_O_5_	[M−H]^−^	269.0450	269.0458	269, 241, 225, 181, 169	[64]
**47**	11.23	Kaempferide	C_16_H_12_O_6_	[M+H]^+^	301.0712	301.0712	301, 286	[73]
**48**	24.77	*δ*- tocotrienol	C_27_H_40_O_2_	[M+H]^+^	397.3107	397.3115	397, 201, 187	[74]
**49**	26.89	*β-* tocotrienol	C_28_H_42_O_2_	[M+H]^+^	411.3263	411.3271	411, 205, 191, 151	[74]

* Interchangeable values.

**Table 3:** IC_50_ values of both *C. annua* crude and phenolics extracts against panel of breast and liver cancer and normal cells using the MTT assay.

Sample	Working Concentrations	IC50 (µg/mL) *				
		Breast			Liver	
		MCF-7	MDA-MB-231	MCF-10A	HepG2	THLE2
*C. annua* crude extract	20, 50, 100, 150, 200 (µg/mL)	22.8 ± 1.01	46.2 ± 1.4	≥50	32.3 ± 1.1	ND
*C. annua* phenolics extract		13.04 ± 0.87	ND	≥50	19.3 ± 0.98	≥50

* Values are expressed as mean ± SD of triplet trials, and calculated using GraphPad prism 7 software using nonlinear regression Dose-Inhibition curve ft.

**Table 4:** Summary of ligand-receptor interactions with binding energy (Kcal/mol) of the identified compounds to “B-cell lymphoma 2 (Bcl-2) protein, PDB = 4IEH).

Compound	Binding Energy (Kcal/mol)	Ligand-Receptor Interactions with the Key Amino Acids	
		HB Interactions	Lipophilic Interactions
Caffeoyl-quinic acid **(6)**	−9.24	1 HB with Arg 66	-
Quercetin-*3-O-*arabinoglucoside **(11)**	−19.30	2 HB with Arg 66 and Tyr 161	-
Quercetin *3-O-β*-D-glucopyranosyl-(1→2)-arabinopyranoside **(12)**	−21.20	2 HB with Arg 66 and Tyr 161	Arene-Cation Arg 66
Kaempferol 3,7diglucoside **(15)**	−18.17	2 HB with Arg 66 and 1 HB with Tyr 161	Arene-Cation Arg 66
Quercetin 3-*O*-[(6-sinapoyl-*β*-glucopyranosyl) -(1→2)-*β*-arabinopyranoside]-7-*O*-*β*-glucopyranoside **(16)**	−27.28	2 HB with Arg 66 and Tyr 161	Arene-Cation Arg 66
Quercetin 3-*O*-[(6-feruloyl-*β*-glucopyranosyl)-(1→2)-*β*-arabinopyranoside]-7-*O*-*β*-glucopyranoside **(17)**	−27.5	2 HB with Arg 66 and Tyr 161	Arene-Cation with Arg 66
Kaempferol-3-rutinoside **(21)**	−18.28	3 HB with Arg 66, 1 HB with Tyr 161	-
Quercetin-4’-glucoside **(23)**	−16.57	1 HB with Arg 66	Arene-Cation Tyr 161
Luteolin-*7-O-*glucoside **(24)**	−21.27	2 HB with Arg 66, and Tyr 161	-
Kaempferol-*3,7-O-bis-α-*L-rhamnoside **(25)**	−23.67	3 HB with Arg 66, 3 HB with Tyr 161	-
Quercetin *3-O-*galactoside **(26)**	−18.17	2 HB with Arg 66 and Tyr 161	-
Quercetin-3-D-xyloside **(27)**	−20.51	1 HB with Tyr 161	Arene-Cation Arg 66
Cyanidin-3-glucoside **(28)**	−18.78	2 HB with Arg 66 and Tyr 161	-
Kaempferol-3-O-glucoside **(29)**	−16.78	2HB with Arg 66	1 arene-cation with Arg 66
Quercetin *3-O*-[(6-sinapoyl-*β*-glucopyranosyl) -(1→2)-*β* arabinopyranoside **(30)**	−15.29	2HB with Arg 66	1 arene-cation with Arg 66

Bolded numbers in parenthesis represent the number of the chemical structure (Figure 4).

## Supplementary Materials

The following are available online at https://www.mdpi.com/2076-3921/9/12/1286/s1; Figure S1. Total ion chromatogram (TIC) recorded in the negative mode for *C. annua* extract; Figure S2. Base peak chromatogram (BPC) recorded in negative ion mode *C. annua* extract; Figure S3. Total ion chromatogram (TIC) recorded in the positive mode for *C. annua* extract; Figure S4. Base peak chromatogram (BPC) recorded in the positive mode for *C. annua* extract.
