# Supplementary material for: Chemical Profiling, Antioxidant, Cytotoxic Activities and Molecular Docking Simulation of Carrichtera annua DC. (Cruciferae)
Source: Antioxidants (Basel). 2020 Dec 16;9(12):1286. doi: 10.3390/antiox9121286 (PMC7766671; doi:10.3390/antiox9121286)
Supplement: Supplementary file 1 [file antioxidants-09-01286-s001.pdf]

Suppplementary Material

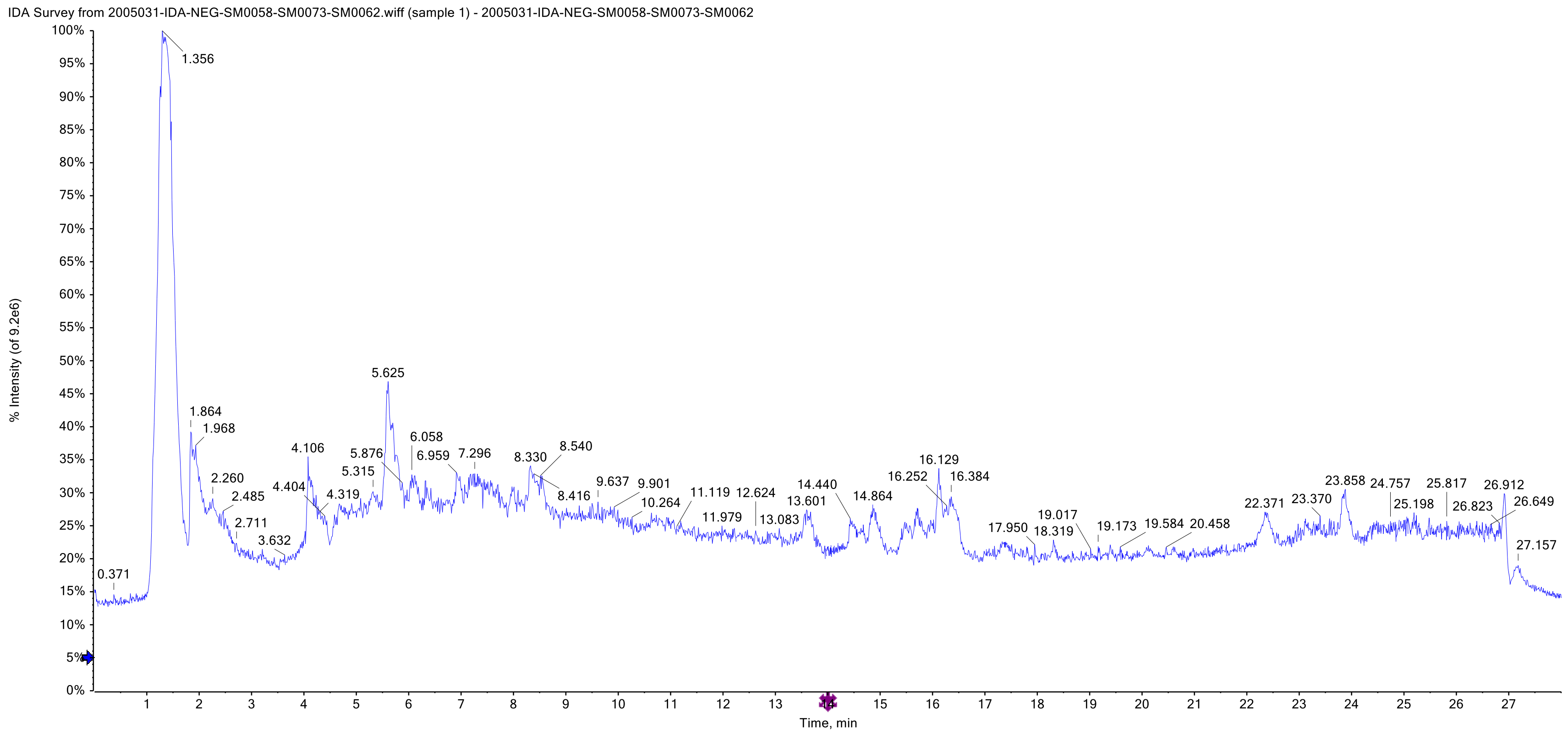

Figure 1. Total ion chromatogram (TIC) recorded in the negative mode for *C annua* extract.

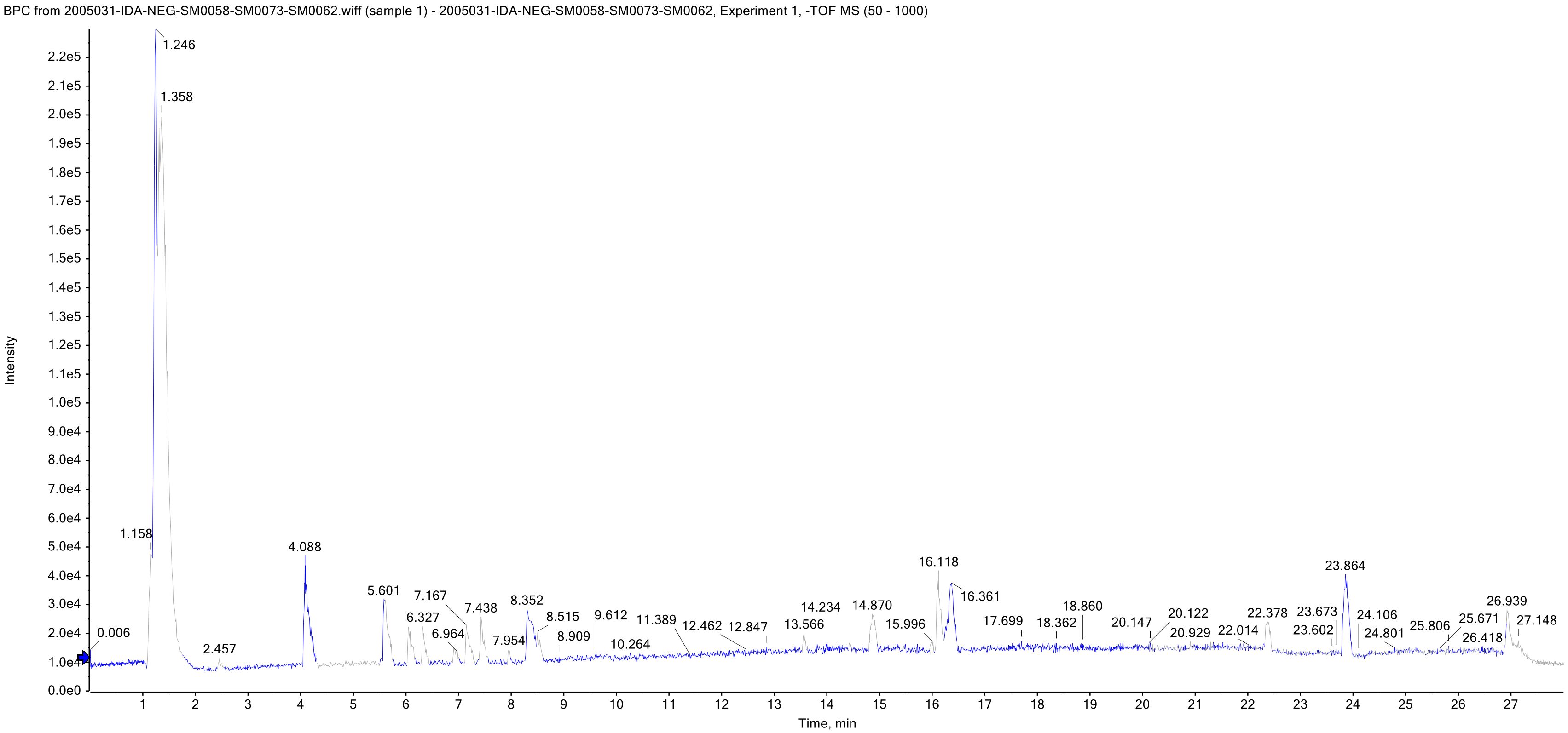

Figure 2. Base peak chromatogram (BPC) recorded in negative ion mode *C. annua* extract.

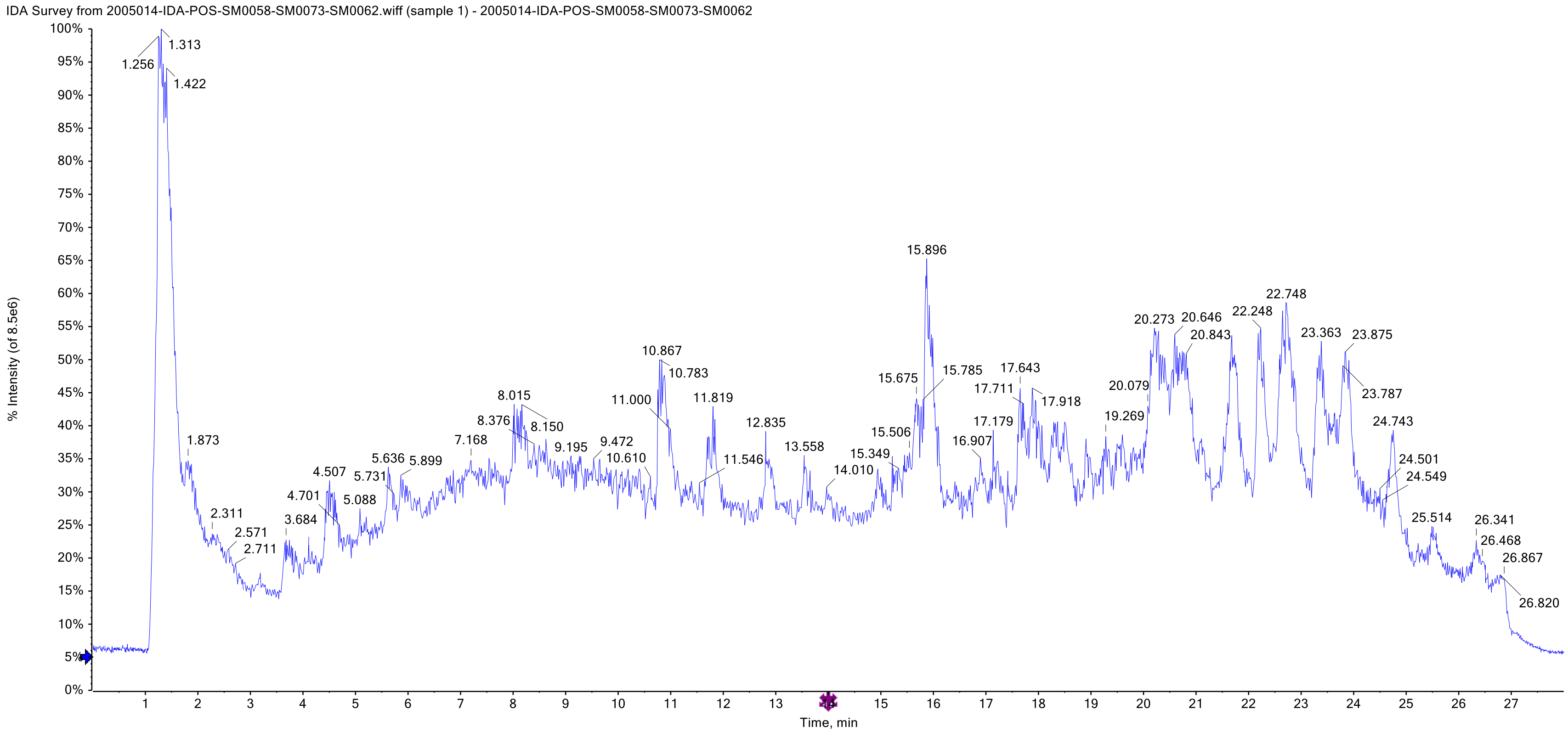

Figure 3. Total ion chromatogram (TIC) recorded in the positive mode for *C annua* extract.

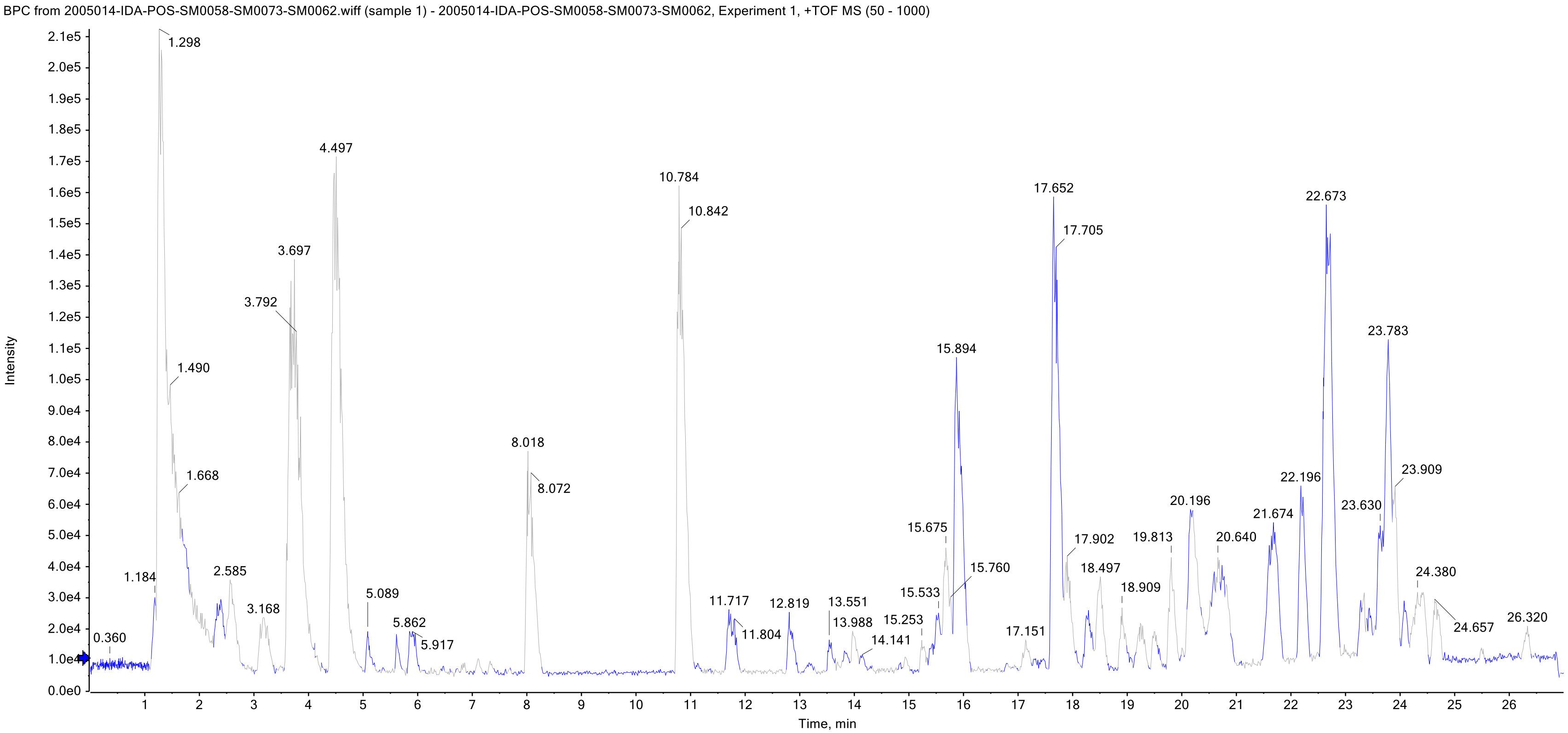

Figure 4. Base peak chromatogram (BPC) recorded in the positive mode for *C annua* extract.
